# Challenging the Top Player: A Preliminary Study on Testosterone Response to An Official Chess Tournament

**DOI:** 10.3390/ijerph17041204

**Published:** 2020-02-13

**Authors:** Guillermo Mendoza, Manuel Jiménez, Jerónimo García-Romero, Jorge García-Bastida, Iván Rivilla, Margarita Carrillo de Albornoz-Gil, Francisco Javier Baron-Lopez, Javier Benítez-Porres, José Ramón Alvero-Cruz

**Affiliations:** 1 Departamento de Fisiología Humana, Histología, Anatomía Patológica y Educación Física y Deportiva, Universidad de Málaga, 29071 Málaga, Spain; gmendoza.t@gmail.com (G.M.); jeronimo@uma.es (J.G.-R.); marcargil@uma.es (M.C.d.A.-G.); benitez@uma.es (J.B.-P.); alvero@uma.es (J.R.A.-C.); 2 Departamento de Didáctica de la Educación Física y Salud, Universidad Internacional de La Rioja, 26006 Logroño, Spain; jorge.garcia.bastida@unir.net (J.G.-B.); ivan.rivilla@unir.net (I.R.); 3 Departamento de Salud Pública y Psiquiatría, Facultad de Ciencias de la Salud-Instituto de Investigaciones Biomédicas de Málaga, Universidad de Málaga, 29071 Málaga, Spain; baron@uma.es

**Keywords:** testosterone, challenge hypothesis, social status seeking, cortisol, ELO rating

## Abstract

According to the Challenge Hypothesis, high levels of testosterone (T) are associated with status-seeking behaviors, especially in competitive situations. However, there have not been many studies about rivals’ social status and pre-competition neuroendocrine responses. The aim of this study was to analyze whether the participants in a chess tournament showed different pre-match testosterone and cortisol levels depending on differences in ELO (i.e., the International Chess Federation rating to rank the competitive potential and social status between players). The sample was six male participants (mean ± SD) aged 25.5 ± 8.4 years with experience in official tournaments of 16.33 ± 5.72 years and an average ELO rating of 2217.67 ± 112.67. Saliva samples were collected before each round for hormonal determination when participants competed against a rival with a different ELO rating. After five competition rounds per participant, higher rival pre-competition T concentrations were shown when playing against the best-rated participant, but there were no differences in cortisol (C). The multilevel model confirmed rises in rivals’ precompetitive T levels modulated by the difference in the opponent’s ELO rating. No significant changes were observed in C. The results suggest that the rival’s status can determine the opponent’s anticipatory neuroendocrine responses to an official chess tournament.

## 1. Introduction

From an evolutionary point of view, reaching a higher social status is strongly associated with access to different resources [1]. Several studies suggest that high testosterone (T) levels are related to a greater interest in reaching and maintaining social status [2]. Many species, including humans, might show fluctuations in T levels in response to social stimuli [3,4]. These fluctuations contribute to better adaptation (e.g., phenotypic plasticity, behavior modification) when facing life events. One of the most widely accepted theories to explain how T fuels competitive behaviors is the “Challenge Hypothesis” [5,6]. According to this hypothesis, T concentrations rise during the breeding season to allow animals to fight for resources. Prior studies suggest that hormone fluctuations are more evident under hierarchical instability [7,8]. Therefore, hormone increase might reveal that an organism is fighting to get resources, reach or defend social status, and maintain dominance. 

Recently, a significant number of studies on sport competition have analyzed neuroendocrine response patterns depending on the final outcome [9]. However, the neuroendocrine response is not just about the final outcome, the personal contribution to winning the match [10], coping with psychological and cognitive variables [11], implicit power motivation [12], or the opponent’s psychological states [13]. In any case, when an organism wins a competition for resources or territory, they are more likely to have an increase in T concentration to allow them to fuel and win future competitive encounters [14,15,16,17]. Some studies have reported the existence of anticipatory responses related to status-seeking behaviors in sports and lab competition tasks. University tennis players as well as judo competitors showed a rise in T before matches, where hormonal and psychological responses were higher before competition compared with during eight resting seasons [14,18]. Professional triathletes showed a similar anticipatory increase in T during competition that was not related to final performance, and the opponent’s psychological state modulated the T anticipatory response to lab competition tasks [19,20,21]. To our knowledge, the Challenge Hypothesis could be one of the most likely explanations to explain the roles of T in official sport competitions, fighting over resources, and status-seeking behaviors [12,19,20,21,22]. Wingfield’s model suggests that a transient increase in T (associated with real competitive challenges) fuels social status-seeking behaviors [23]. However, T not only plays a relevant role in competitive behaviors and social dominance, cortisol (C) is also important to mobilize glucose to the skeletal muscle in order to get the body ready to fight or flee. High C levels can boost or reverse the effect of T in status-seeking or status-maintaining behaviors [24,25], and there are several interactions between T and other hormones and catecholamines [26,27].

The neuroendocrine response patterns to sport competition and, more specifically, in chess tournaments have already been researched. Mazur, Booth and Dabbs [28], analyzed T levels among 16 chess players, categorizing the statuses of the participants into two groups: “winners” and “losers”. Their results concluded that winners presented higher T levels after several victories in two tournaments of different degrees of difficulty, suggesting that chess could be an excellent field of study for the subjacent mechanisms of the T-dominance relation, mainly because it provides the possibility to categorize a social-status rank following standardized and worldwide-accepted systems that reflect a player’s hierarchical position and real competitiveness (i.e., ELO rating [29]). Mazur et al. [28] argued the fact that having the possibility to predict the skill level of the following opponent (e.g., ELO rating) could be a possible explanation for why losers presented higher initial T levels than winners. Studies have observed the same pre-competitive response of losers in many other sports [10,14,15,16,24]. As far as team sports are concerned, a significant increase in pre-competition T levels when rivalry between opponents is perceived as extreme has been shown [30]. 

Consistent with the Challenge Hypothesis, the dynamic relation between T and competitiveness in a status-seeking context would increase the hormone levels when facing a major challenge. Accordingly, in a chess tournament, the lower-rated participants would presumably be strongly motivated to beat an opponent who has a higher ELO rating. Thus, T levels would get readjusted depending on the intensity of the challenge. The aim was tested with a preliminary study to analyze whether chess players showed any T and C differences related to precompetitive response or time spent on opening moves (i.e., starting a strategy in the first ten moves) when playing against a higher-rated opponent.

## 2. Materials and Methods

### 2.1. Participants

The sample was composed of six male participants of Spanish and French nationality; all of them were active chess players and members of the Andalusian Chess Federation (FADA). Their characteristics were as follows (mean ± SD): age = 25.5 ± 8.4 years; experience in official tournaments = 16.33 ± 5.72 years; ELO rating = 2217.67 ± 112.67. All participants had the level of “Expert” or “FIDE Master” recognition according to their ELO rating [26] and were therefore considered to be at the highest level of competitive chess. Among the participants, only the highest rated player was conceded an extra status distinction. He was the local champion, the highest ELO-rated player, and all the opponents recognized him as the most likely to win the tournament.

### 2.2. Procedure

The rules used for the participants were the same as for an official tournament and, therefore, were applicable for the ELO rating. The time setting was 60 min per side plus an additional 30 s for every move. It was a two-day tournament: the first three rounds took place on the first day (from 9 a.m. to 9 p.m.) and the last two were played on the following day (from 9 a.m. to 2 p.m.). In order to promote competitiveness and commitment, prizes worth 200€ were offered for the winners. Previous studies [28] suggested a major limitation in their game matching system was that the players who won more frequently were also those who competed against the weakest rivals. To avoid the possible effects that this variable could have on the results, the present study used a competition system in which each participant played against all other contestants in turn, up to a total of 15 games. The “round-robin” system has the ability to balance the difficulty that every player would face and allows one to organize all rounds prior to starting the competition. Players alternated white and black pieces in each round so as to minimize the possible advantage that one or another could mean for the results. To organize the first round, participants were sorted according to their ELO FIDE rating (i.e., their competitive potential and social status in this sport). The highest ELO-rated participant was referred to as J1 (considered the top player); the second highest ELO-rated player was named J2, and so on until J6. This rating is a four-digit number that represents the skills of each player, regulated by International Chess Federation (FIDE). Gaining hierarchy in this sport depends on both match results and the ELO rating of each opponent. Thus, a draw against a higher-rated player could lead to a major increase in the ELO rating compared with beating a lower-rated opponent. This system is considered to be an optimal indicator of the competitive potential and makes it easier to identify the best players. In addition, it is considered the primary pairing criterion in official tournaments, as it reliably determines the status of each player in relation to the opponent. Defeating a higher ELO-rated opponent not only takes the player to a higher social status, but it also implies having greater access to economic opportunities and sponsorship as well as causing a remarkable impact on their social circle. The bio-social model of status also suggests that facing a higher-ranked opponent and failures in the past could be associated with decreases in precompetitive T [14]. To minimize this variable influence on T, players were selected between the highest percentiles. All chess players were ranked over the 85th percentile in the Spanish Federation’s Rank; hence, they competed frequently to be among the top ten in prior tournaments, had previous experience winning and losing against others, and were willing to be the victorious player. The tournament was performed in a neutral territory to reduce the “home advantage” [30,31,32].

The participants were instructed not to drink water, eat, or brush their teeth within the 15–30 min prior to the sample collection. Medical reports were collected so as to dismiss endocrine, psychological, physiological, or drug disorders; then, all participants signed a consent form. T and C levels were obtained by means of a saliva test (5–8 mL). Standard plastic tubes were given so as to obtain saliva samples for the immunoassay (Salivette®, Sarstedt, Alemania). Each round took about 90 to 120 min. From the end of the previous match until next one, players were allowed to have a walk around the hall or street and drink or eat something for more than 30 min. After this time, they waited without eating, drinking, or brushing their teeth for 30 to 50 min; this waiting time (between 70 to 90 min) was designed to reduce the influence of the winning effect on T. Samples were collected 5 min prior to the beginning of each round (at 9 a.m., and 12, 3, and 6 p.m.), then refrigerated at 5 ºC, and stored at −40 ºC until the immunoassay. Samples were centrifuged at 3000 rpm and immunoassayed in the hematology laboratory at the Virgen de la Victoria hospital in Málaga using Grifols Triturus^®^ equipment (Grifols, Barcelona, Spain) and enzyme-linked inmunoabsorbent assay (Diametra^®^, Milano, Italia) with intra and inter assay coefficients for T and C of 10.1 and 7.9% and 6.8 and 4.2% with sensitivity levels of 3.28 pg/mL and 0.5 ng/mL, respectively. The detection limit was 0 to 1000 pg/mL for T and 0 to 100 ng/mL for C. Samples were assayed twice.

The minutes (M) that each participant spent on the first ten moves were annotated. In chess, the first moves are called “openings” and are one of the most important parts of the game. They consist of setting the pieces strategically to define the game plan. Experienced players prepare the openings that they will use against each opponent in advance, so that they can save time in this first part of the game. The time spent by each player on these first moves might be an indicator of a cautious competitive behavior or of how confident—or not—they are with a strategy chosen against one specific opponent. Once the game was over, a copy of the movement sheets was collected and then transcribed into the open source chess engine Stockfish 21 (licensed under GPL license3.0) to determine the number of errors (E) that each player made in each round [33]. Moves that changed the evaluation numbers given by Stockfish by one point or more were considered an error [34]. This study was approved by the University of Malaga Ethical Committee with code CEUMA 26-2018-H.

### 2.3. Statistical Analysis

The collected data were organized according to the rival against whom each participant played in each round. As participants could not play against themselves, this value was completed with the mean of the group, in line with the procedures used in previous investigations [25]. According to the steps set out in these studies, in order to minimize the individual variations among the subjects, values were divided by the highest one. As a result, data were normalized by values between 1 and 0 [28,35] and named as follows: T_1_ for the normalized mean values of T when playing against J1, T_2_ for the normalized mean values of T when playing against J2, and so on until T_6_ for the mean values of T when playing against J6. The Wilcoxon signed rank test (and the Cohen’s d) was performed to assess the differences when comparing the number of errors and the time spent on the openings against each participant. Multilevel models were also performed to examine whether T and C concentrations interacted with the difELO between players (T ∼ difELO + (1 player) and C ∼ difELO + (1 player)) or between players and the round played (T ∼ Round+player + difELO:Player and C ∼ Round+player + difELO:Player), respectively. Confidence intervals and R-square coefficients of determination for each player were also determined. Linear mixed-effects models were fitted using the library lme4 model [36] from R. The post-hoc power calculations were computed using the “simr package” [37], which uses previously fitted lme4 models and Monte Carlo simulations to run the power analysis for a given model and design. Results were considered to be significant at *p* < 0.05.

## 3. Results

**Figure 1:**
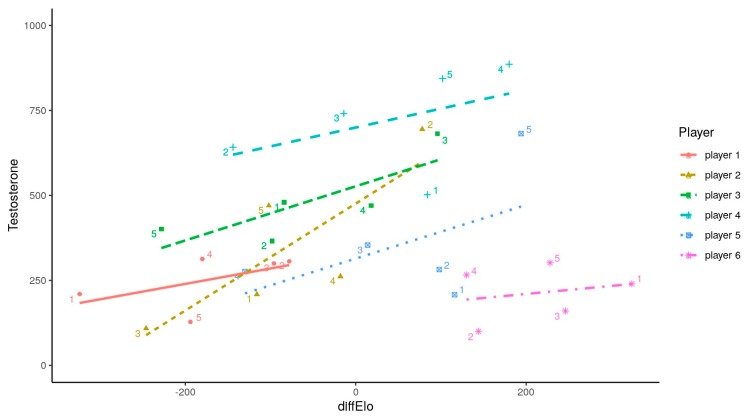
Individual salivary testosterone (pg/mL) production related to opponent’s ELO difference and number for each round. difELO = ELO difference between opponents; J1 = player 1; J2 = player 2; J3 = player 3; J4 = player 4; J5 = player 5; J6 = player 6.

**Figure 2:**
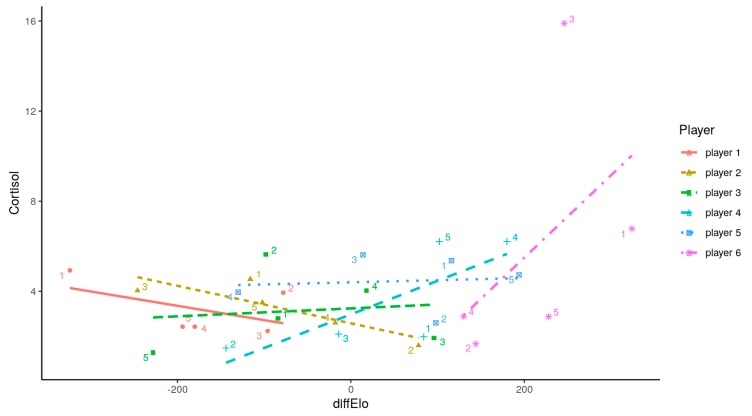
Individual salivary cortisol (ng/mL) production related to opponent’s ELO difference and number of each round. difELO = ELO difference between opponents; J1 = player 1; J2 = player 2; J3 = player 3; J4 = player 4; J5 = player 5; J6 = player 6.

Figure 1 shows the multilevel model of the difference in difELO and its influence on the opponent’s hormonal response to competition; the difELO rating was related to the opponent’s precompetition T concentration (*t* = 3.307, *p* < 0.01) but was not related to C (Figure 2)

Table 1 shows the mean number of minutes that each player spent on the first moves and the errors that they made per round against each rival. The planned strategies, as well as the pressure that the competitive level of the rival puts on the player, could be important factors to take into account. However, statistical significance was only found when comparing the time spent against J1 with the time spent against J2 and J6 (z = −1.992, *p* < 0.046 and z = −1.992, *p* < 0.046, respectively). Lacking a consistent statistical difference, the number of errors was lower in gross terms but not statistically significant.

Table 2 and Table 3 show multilevel models. Bigger difELO values modulated rises in T concentrations (*t* = 5509, *p* < 0.001). Hormonal fluctuations were affected by each round (*t* = 3229, *p* < 0.01). No correlations between individual hormonal changes were related to winning/losing the previous match (*p* > 0.11), suggesting that fluctuations could not be affected by results. Table 2 shows individual testosterone changes depending on differences between rivals in ELO rating (difELO) with 95% CI and coefficient of determination (i.e., R^2^). Table 3 shows the multi-level model and differences in ELO rating between rivals (difELO) and the round played with 95% CI and coefficient of determination.

The post hoc bootstrap/Monte Carlo simulation to minimize the type II error showed a power effect of 1 – β over 0.90 for the multi-level statistical analysis.

## 4. Discussion

The aim of the present study was to identify possible differences in pre-round T and C concentrations in male chess players and probably in the decision-making process during the tournament depending on the rival’s ELO rating. Pre-round T concentrations were significantly higher when the participants played against players with a bigger difELO, but there were no differences in C concentration. Consistent with the Challenge Hypothesis, results indicated that the rival’s status could be a modulating factor in T production and probably in decision-making in competition contexts, suggesting that different strengths, human competitive behaviors, and a player’s social hierarchy could affect their rival’s hormonal concentrations.

Previous investigations by Mazur about chess [28] revealed that losers showed higher T levels than winners before a tournament. They argued that losers could have perceived in advance who their rivals would be, thus suggesting the existence of an anticipatory response. This explanation is based on the use of the “Swiss system” to organize the players who would compete in each round which allows players to know beforehand that the first opponent of the tournament would be an extreme challenge for the lower-rated players. The present study, ignoring this limitation, used the “round-robin” system to pair the rivals, as it necessitates playing against all participants in successive rounds. The most important thing about this experimental outline of successive rounds was that the difference in the strength of the rivals was balanced.

Following the Challenge Hypothesis, the T level increases in response to a fight over resources, especially when social hierarchy is unstable or has been challenged [7]. In the original Wingfield’s Model, the effects of the “challenge” were modulated by the status gradient between rivals and behaviors induced by T. However, professional sports competitions and some other some human social interactions could be equivalent to a challenge situation: "studies involving non-physical competitive situations seem produce comparable results to sports" [7]. In this study, T concentrations changed at the beginning of each round, and a greater rise was observed when the participants played against the best-rated opponent in a chess tournament (i.e., with a bigger difELO). This peak could have been caused by the economic opportunities for the final winner (i.e., appetitive and tangible reinforcement). The effect of the economic prize as reinforcement has been studied in several investigations as a behavior stimulus focused on the objective [38,39,40] and has also been related to a rise in T to be more competitive and get the resources [35]. In addition to economic opportunities being present in the competition, winning or playing to a draw with a higher-rated opponent meant more ELO points than beating a lower-rated participant. Defeating a higher-rated player caused a significant boost in the individual ELO rating, consequently improving the social status of the participant within the group. “Challenging” the top-rated opponents probably provided a valuable incentive, meaning direct access to more prestigious competitions and the possibility of getting more prestigious chess titles. Hence, the neuroendocrine response observed in each round fitted perfectly with that suggested in the Challenge Hypothesis. According to this theory, in competitive situations, androgen release increases when hierarchy and/or the access to resources has been challenged [5,6]. Androgen production is not systematically observed in every human interaction; higher T concentration levels coincide with the most extreme challenges [8]. Thus, a bigger difELO between rivals (i.e., when greater number of ELO points was in play and there was a higher probability of climbing in the ranking) produced higher rises in pre-competitive T. Direct competition could be a vast field to observe anticipatory responses consistent with the Challenge Hypothesis [13,38,41] in sport competition [9]. Regardless of the lack of physical effort, the T rises observed have a good preliminary foundation to fit with Wingfield’s model and suggest that real sport competition is an excellent field to research social neuroendocrinology in humans (as argued by Wingfield [6]). This study design must be repeated in additional tournaments with a larger sample size to allow generalization; our preliminary results suggest that an anticipatory T response occurs when professional players challenge the top player.

However, in Mazur’s study, when there was a difference of over 200 ELO points between rivals, no significant changes in T were observed for victorious or defeated players [25]. Following previous results, it is important to highlight that J6 did not show a significant increase in T concentration when playing against J1, nor against J2. However, the multilevel model showed that fluctuations in T concentrations were generally linked with difELO (see Figure 1) in J6. This player presented higher concentrations when playing against competitors with the nearest difELO. The perceived rivalry could be an important modulator of competitive behaviors [27], boosting interest in winning direct fights so as to keep or seek a better social status when this option was real.

Not only high concentrations of T affect status-seeking behaviors, but also, C concentrations play a key role [42]. After analyzing the fluctuations in this glucocorticoid, the conclusion is that no significant differences were observed when the participants played against J1 compared to when they competed against the rest of the rivals. The special characteristics of this sport (i.e., lack of physical activity) exclude the effect that the intensity of physical exercise could have on the production of this hormone [43]. Laboratory studies showed that cognitive tasks, mostly when these are constant and assessed by a third person, produce a sharp C peak [44]. However, our results did not show this tendency in chess competition. A possible explanation for this could be the link between a better performance in mental tasks and attention skills only when C production is moderate [45,46]. Participants were highly experienced players in official competitions and had consequently mastered relevant knowledge of the conditions to achieve optimal individual performance [47]. Likewise, previous experience of anticipating a chess tournament did not contain the factors necessary for eliciting a cortisol response [23]. The struggle to control pre-competition stress could be an important factor in determining the final outcome. Another explanation of the observed fluctuations could be the circadian rhythm. This means that there are higher concentrations in the morning that progressively decrease as the day goes by, reaching their lowest peak at the end of the day [48]. However, C production stability on both days of the competition, regardless of the time of the day when samples were collected, suggests that the influence of the circadian rhythm on the hormone response patterns was limited. To look at how each round could affect hormonal concentrations (i.e., the influence of circadian cycles, because round 1 was at 9 a.m., round 2 was at 12 p.m., round 3 was at 3 p.m., and round 4 was at 6 p.m.), a multilevel model was performed to confirm whether rounds played could be an important variable to be consider in players’ hormonal responses. Rounds modulated the hormonal production, but their influence reduced the error on T changes related with difELO. To allow a better understanding, Figure 1 and Figure 2 show the round numbers for each player, but the most important competition hormonal response was still playing against players with higher difELO.

During the chess games, there could have been indicators of interest and effort in defeating the highest-rated player. This interest was observed in the minutes spent on the openings against J1. The first moves (i.e., openings) reveal the prudence or impulsiveness of the player when preparing the game strategy. There were no differences when players fought against J1. Nonetheless, it is important to highlight the tendency of spending more minutes when competing against the favorite player (mean time: 19.4 min). Furthermore, when the number of errors was analyzed, no significant differences were observed related to the rival’s hierarchy, but there was evidence of a trend of making more errors against J1. A better prepared strategy and effort to make fewer errors are both coherent behaviors with a greater interest in defeating the highest ELO-rated player. However, this study could not find evidence for conservative or risky playing patterns depending on rival status. 

## 5. Limitations

Some limitations present in this investigation must be considered. First, the sample was just six participants with 30 observations. Official tournaments commonly have a reduced number of participants playing in the round-robin system, which was essential for the study design. Nevertheless, it is considered necessary to design an experiment with a greater sample to confirm or refute this preliminary study [46,49]. Multilevel statistics confirmed that a higher anticipatory T concentration was related to a bigger difELO between players, but there were no effects on C. A second limitation was that the rounds were played at different times of the day with a crucial consequent effect of the circadian rhythms on hormone concentrations. Previous investigations pointed to circadian rhythms being closely related to hormonal fluctuations throughout the day [50] and also with making decisions [31]. Hence, it is important to highlight that the salivary concentrations of T and C did not decrease during the competition. The third limitation to consider was that participants played against each other (i.e., they did not start from a base point in each game). The multilevel model showed interactions between rounds and T, suggesting that the number of rounds played influenced the T response, but the confidence interval coefficient just varied by less than 10% with respect to that obtained when the influence of the round was not analyzed. All participants were males, and this important gender-related limitation must be addressed in future studies; many researchers are interested to know how hormonal fluctuations affect women when they face a high-ranked adversary [51].

## 6. Conclusions

Different anticipatory neuroendocrine responses were observed depending on the status of the rival. Fighting to defeat a player with a higher hierarchy produced a rise in the pre-competition T concentration, which is consistent with the Challenge Hypothesis. These anticipatory neuroendocrine response patterns are congruent with status-seeking behaviors. When players faced rivals with a higher difELO, they had a bigger T increase, suggesting a new explanation for the mechanisms underlying the hormonal response in chess players. The tendencies observed for the time spent setting the strategies as well as the lower number of errors made against the highest-rated participant need further investigation in future research. 

## Figures and Tables

**Table 1:** Mean ± standard deviation (SD) for minutes spent on the openings and errors per round against each rival.

	M_1_	M_2_	M_3_	M_4_	M_5_	M_6_
Mean	19.4	4.8 *	12.4	11.8	8.2	6.6 *
SD	9.65	4.96	11.16	6.14	7.88	3.2
	E_1_	E_2_	E_3_	E_4_	E_5_	E_6_
Mean	0.8	2	3.2	2.6	1.4	2
SD	0.75	1.79	3.31	1.62	1.2	3.03

M_1_ = mean value of the minutes spent on the first 10 movements against J1; M_2_ = mean value of the minutes spent on the first 10 movements against J2; M_3_ = mean value of the minutes spent on the first 10 movements against J3; M_4_ = mean value of the minutes spent on the first 10 movements against J4; M_5_ = mean value of the minutes spent on the first 10 movements against J5; M_6_ = mean value of the minutes spent on the first 10 movements against J6. E_1_ = mean value of the errors made in the match against J1; E_2_ = mean value of the errors made in the match against J2; E_3_ = mean value of the errors made in the match against J3; E_4_ = mean value of the errors made in the match against J4; E_5_ = mean value of the errors made in the match against J5, E_6_ = mean value of the errors made in the match against J6. * *p* < 0.05 M_1_ vs. M_2_ and M_1_ vs. M_6_

**Table 2:** Confidence intervals (CI) for individual testosterone changes depending on ELO differences between rivals.

Predictors	Estimates	CI 95%
Player 1	0.45	−0.88 to 1.79
Player 2	1.57	0.49 to 2.66
Player 3	0.80	−0.27 to 1.86
Player 4	0.56	−0.49 to 1.60
Player 5	0.79	−0.27 to 1.85
Player 6	0.24	−1.4 to 1.88
Observations	30	
R^2^	0.78	

ELO = Player’s Chess Federation rating, R^2^ = determination coefficient.

**Table 3:** Multi-level model depending on ELO differences between rivals and round played.

Predictors	Estimates	Standard Error	CI 95%	*p*-Value
(Intercept)	294.30	98.66	100.93–486.67	0.003
Round	33.97	15.14	4.28–63.65	0.025
DiffELO	0.74	0.20	0.34–1.13	<0.001
		**Random Effects**		
σ^2^		13762.24		
T_00 Player_		43263.79		
ICC		0.76		
N _Player_		6		
Observations	30		
R^2^		0.813		

DiffELO = ELO Rating differences between players; R^2^ = determination coefficient; σ^2^ = CI Variance; ICC = intraclass correlation; CI = Confidence Interval; N = number of participants. T_00 Player_ = variance of player’s random intercept.
